# Infrared Thermal Imaging as a Novel Non-Invasive Point-of-Care Tool to Assess Filarial Lymphoedema

**DOI:** 10.3390/jcm10112301

**Published:** 2021-05-25

**Authors:** Louise A. Kelly-Hope, Mohammad Jahirul Karim, ASM Sultan Mahmood, Abdullah Al Kawsar, Abul Khair, Hannah Betts, Janet Douglass, Armelle Forrer, Mark J. Taylor

**Affiliations:** 1 Centre for Neglected Tropical Diseases, Department of Tropical Disease Biology, Liverpool School of Tropical Medicine (LSTM), Liverpool L3 5QA, UK; hannah.betts@lstmed.ac.uk (H.B.); jan.douglass@jcu.edu.au (J.D.); Armelle.Forrer@lstmed.ac.uk (A.F.); mark.taylor@lstmed.ac.uk (M.J.T.); 2 Filariasis Elimination, STH Control and Little Doctor Programme, CDC, DGHS, Ministry of Health and Family Welfare, Dhaka 1000, Bangladesh; jahirulkarim@gmail.com (M.J.K.); syeed25@gmail.com (A.S.M.); drakawsar@gmail.com (A.A.K.); abulkhair018@gmail.com (A.K.)

**Keywords:** infrared thermal imaging, lymphatic filariasis, lymphoedema, NTDs, neglected tropical diseases, skin temperature, tissue tonometry, point of care

## Abstract

Lymphatic filariasis causes disfiguring and disabling lymphoedema, which is commonly and frequently exacerbated by acute dermatolymphangioadenitis (ADLA). Affected people require long-term care and monitoring but health workers lack objective assessment tools. We examine the use of an infrared thermal imaging camera as a novel non-invasive point-of-care tool for filarial lower-limb lymphoedema in 153 affected adults from a highly endemic area of Bangladesh. Temperature differences by lymphoedema stage (mild, moderate, severe) and ADLA history were visualised and quantified using descriptive statistics and regression models. Temperatures were found to increase by severity and captured subclinical differences between no lymphoedema and mild lymphoedema, and differences between moderate and severe stages. Toes and ankle temperatures detected significant differences between all stages other than between mild and moderate stages. Significantly higher temperatures, best captured by heel and calf measures, were found in participants with a history of ADLA, compared to participants who never had ADLA, regardless of the lymphoedema stage. This novel tool has great potential to be used by health workers to detect subclinical cases, predict progression of disease and ADLA status, and monitor pathological tissue changes and stage severity following enhanced care packages or other interventions in people affected by lymphoedema.

## 1. Introduction

Lymphatic filariasis (LF) is a parasitic neglected tropical disease (NTD) targeted for global elimination [1]. Infection drives inflammatory immune responses, which can impair lymphatic function, leading to pathological tissue change [2]. There are two main sequelae, hydrocoele (scrotal swelling; affecting approximately 25 million men), and lymphoedema (skin/tissue thickening; affecting approximately 15 million people). These chronic disabling and disfiguring conditions can be exacerbated by secondary bacterial infections, causing acute inflammation or acute dermatolymphangioadenitis (ADLA) [3,4]. These conditions have a significant impact on the physical, social, mental and economic well-being of affected people and the family members who care for them [4,5,6]. Even when transmission of infection is interrupted through mass drug administration, the need to provide life-long care to those with clinical presentation remains.

The World Health Organization (WHO) advocates for managing morbidity and disability prevention (MMDP) through an essential package of care as part of the current WHO NTD Road Map for 2021–2030 [1]. However, MMDP activities lag behind targets set for the drug distribution aspects of the programme, and this is partly due to the difficulty in collecting objective measurement data in endemic areas. Furthermore, people affected by lymphoedema require long-term care and there is a need to improve capacity and integrate services into primary health care as part of universal health coverage. In order to achieve this, we need effective and inexpensive tools for researchers to provide the evidence for recommended practices. Such tools must also be accessible to health workers so they can monitor progress among existing cases, and detect new subclinical cases for preventive intervention [7,8,9,10].

Filarial lymphoedema is most commonly measured and monitored using clinical staging systems, based on limb size and skin conditions. This helps to categorize severity and progression of disease but criteria across systems are inconsistent and subject to observer bias [3,11,12]. Device-based attempts to collect objective measures in low-resource settings have included a bucket of water to estimate limb volume, and a tape measure to determine limb circumference [13,14]. These measures can quantify limb size but fail to capture other clinically relevant staging criteria. Tissue tonometry has been used in research on other forms of lymphoedema to quantify changes in skin stiffness and tissue compressibility. Good intra-rater reliability at all stages of filarial lymphoedema [15] including subclinical tissue change has been reported [9], but it is not widely available or accessible to local health staff. More recently, a portable three-dimensional infrared imaging tool has provided an innovative way to estimate limb volume measurements in a field setting [16,17].

Infrared thermal imaging presents an innovative and objective method for quantifying clinical change in lymphoedema status by using naturally emitting infrared radiation to capture skin surface temperatures [18,19,20,21]. This may be a useful measure of chronic inflammation associated with a history of ADLA [22], and a simple way to objectively monitor progression of disease or detect new cases in the field. Infrared thermal imaging has been used for more than 50 years in a wide range of medical disciplines [18,19,20], including vascular disease [23], skin and tissue conditions [24,25,26], and recently for snakebite envenoming [27]. There are a range of compact thermal imaging cameras available, at relatively low cost and which would be suitable for use by field researchers and local health workers after minimal training, as an objective measure of filarial lymphoedema.

The aim of this study was to examine the use of an infrared thermal imaging camera as a novel non-invasive point-of-care assessment tool for filarial lymphoedema in an endemic area of Bangladesh. The specific objectives were to visualise and quantify temperatures by lymphoedema stage and ADLA history at selected regions of interest (ROIs) on the lower limb, and to examine the relationship between two new objective measurement tools, including thermal imaging and tissue compressibility. Results will further our understanding of the pathogenesis of filarial lymphoedema, and have the potential to provide empirical evidence to inform best practice recommendations for lymphoedema management and clinical interventions in LF-endemic countries.

## 2. Materials and Methods

### 2.1. Study Site and Participants

Bangladesh has one of the highest LF morbidity rates in the world, and extensive data on cases. A cross-sectional study was conducted in October 2020 in the highly endemic district of Lalmonirhat in the northern Rangpur Division of Bangladesh, where patient searching surveys conducted in 2013 found an estimated 5383 people with lymphoedema (male 1077; female 4306) and a prevalence rate of 418.4 per 100,000 population [28].

Surveys to collect personal data, medical and lymphoedema history were conducted in 15 community clinics across three subdistricts, Aditmari, Kaligani and Sadar Upazilas, coinciding with a defined lymphoedema hotspot [28]. Community clinic patient registers were used to identify and invite people affected by lower-limb lymphoedema to participate in a study on lymphoedema self-care.

Inclusion criteria were ≥18 years of age, able to provide informed consent and perform self-care independently or with a carer once trained. Exclusion criteria were conditions that could impact limb lymphoedema and temperature and bias the results; having an ADLA at the time of survey, an injury to either leg, surgery for lymphoedema, too unwell to participate, febrile, known history of heart or kidney disease, diabetes, and hypertension.

Participants were asked to arrive at the clinic early so they could be pre-screened by the field team to ensure they had lymphoedema in one or both legs, and so that all surveys could be conducted over a few hours in the morning usually between 09.30 and 12.00 and not later than 14.00 to avoid variation in fluid accumulation that typically presents towards the end of the day. This helped to control for any variability in participant pre-survey physical activity and daytime ambient temperature, which may affect outcome measures [17,21,29,30,31,32].

Participant information included age, sex, marital, education and employment status. All participants were provided with information on LF and training on an enhanced self-care protocol for lymphoedema management after the survey [31,33].

### 2.2. Lymphoedema Stage

Participants’ legs were assessed by experienced field teams. First each leg was graded using the Dreyer seven-stage classification system [12] and then re-grouped into simplified staging of lymphoedema broadly in line with the WHO staging criteria [3,11];

(i) No lymphoedema (Stage 0);
(ii) Mild lymphoedema (Stage 1), variable swelling may reduce on elevation or overnight, no skin changes;
(iii) Moderate lymphoedema (Stage 2), permanent shallow skin folds, irreversible limb enlargement, presence of minor skin changes such as small knobs;
(iv) Severe lymphoedema (Stage 3), permanent deep skin folds, pathological skin changes including large knobs, sclerosis, discolouration and/or mossy lesions (papillomatosis).

Participants’ reported ADLA history was included as: had ever/had never experienced ADLA, number of ADLA episodes in the past month, and past six months. To minimise self-reported and recall biases, all participants were questioned in the same way.

### 2.3. Temperature and Regions of Interest (ROIs)

Temperature measures of the legs were captured using the FLIR C3 Compact Thermal Imaging Camera [34], which has a 128 × 96 pixel resolution with 70 mK thermal sensitivity, and can detect and measure temperature between −20 degrees and +300 degrees Celsius (°C) to an accuracy of ±3 °C/3%. The FLIR C3 built-in digital 5 megapixel camera was used to collect a standard digital photograph, and its Multi-Spectral Dynamic Imaging (MSX) enhancement setting was used to optimise images. The emissivity, a measure for quantifying the effectiveness of a surface radiating energy ranging from 0 (low) to 1 (high), was set in the camera at 0.95 as skin is a strong emitter of thermal radiation [21].

Three images of the participant’s bare lower legs (standing, feet 30 cm apart) were taken in a private location in the community clinic. The camera was set on a tripod approximately 0.5 m from the ground, and 1 m from the participant for the whole lower-leg images (front and back), and a close-up image of the ankle and toes was taken from, approximately 0.5 m above the foot at a 45 degree angle. The camera took 1–2 min to calibrate and the images for each participant between 10 and 15 min depending on their level of disability. An example of the camera set up is presented in online Figure S1.

**Figure 1:**
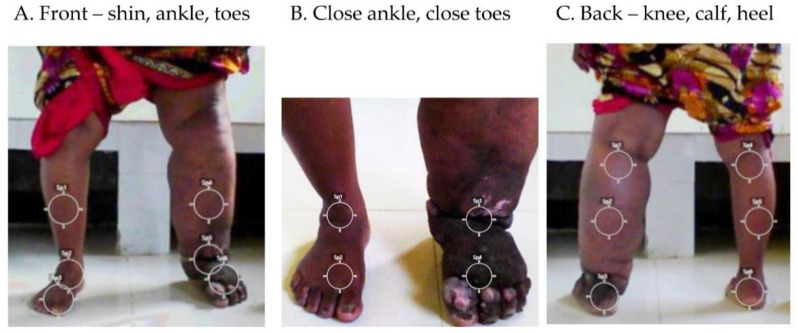
Location of temperature regions of interest (ROI) on front and back of legs. In total eight ROI on each leg were selected and included (**A**) the anterior leg at shin, ankle and toes on the whole-leg at 1m distance; (**B**) close-up of ankle and toes at 0.5 m distance; (**C**) the posterior leg at the back of the knee, calf and heel on the whole-leg at 1m distance.

Eight ROIs on each leg were selected for temperature image analysis. There were five ROIs on the anterior leg at shin, ankle and toes on the whole-leg images (Figure 1A), and ankle and toes on the closer-up images (Figure 1B), and three ROIs on the posterior leg at the back of the knee, calf and heel (Figure 1C). The camera angles and ROIs were selected to provide a complete image of the affected areas in order to align temperature data with the measurement points used for limb circumference and tissue tonometry measures at the mid-calf, and to capture regions of potential infection such as the interdigital spaces and within shallow or deep skin folds.

### 2.4. Objective Limb Measurements

Two objective measurements of lymphoedema status were collected at mid-calf. First, the circumference of each leg was measured in centimetres (cm) using a retractable tape measure at the mid-point between the heel and the knee [13]. Second, the hand-held Indurometer (BME-1563; Flinders and SA Biomedical Engineering, Bedford Park, Australia), which is an electro mechanical tissue tonometer, was used to measure tissue compressibility or stiffness of the mid-calf [9,32]. Briefly, the Indurometer has an indenter which is pressed gently into the skin and underlying tissue and records the distance the indenter has been able to indent the tissue in millimetres. A lower value indicates more tissue stiffness (less compressible), and more compressible tissue (less stiff) will return a higher value [9,35].

### 2.5. Room Measures

To determine or control for the effect of ambient conditions on thermal images, data on temperature and humidity were recorded in each community clinic using a portable ThermoPro TP49 Digital Room Thermometer Indoor Hygrometer, which had a temperature ranges from −50 to 70 °C and humidity ranges from 10 to 99% relative humidity.

### 2.6. Data Collection, Visualisation and Analysis

Survey data were collected using the Open Data Kit Collect (ODK Collect) application loaded on two electronic tablets (Samsung Galaxy 10), downloaded as a CSV and imported into STATA version 15.0 (StataCorp LP; College Station, TX, USA) for descriptive and statistical analysis. The ODK parameters were set, so all questions were answered to ensure there were no missing data.

To visualise temperature differences by lymphoedema stage, digital images taken with the thermal imaging camera were imported into the FLIR Tools software [34]. The thermal image colour and temperature scale were adjusted for optimisation. The average temperature from each of the eight ROIs were quantified for each patient. For this paper, four participants affected by lymphoedema at different stages were selected as case studies, and demographic, lymphoedema stage, ADLA information, leg temperatures, leg circumference, calf compressibility, room temperature and humidity were summarised.

To quantify temperature differences by lymphoedema stage and ADLA history, temperatures from the eight ROIs on each patient were entered in the survey database. Regression models and analyses were performed in STATA. Two series of mixed-effects linear regression models were run. The first aimed at estimating temperature differences by lymphoedema stage and the second at assessing differences in temperature, by lymphoedema stage in presence or absence of ADLA history. All models included individual-level and village-level random effects assuming an exchangeable correlation structure. For the variable selection, mixed-effects linear regression models, adjusting for room temperature were used. The association between temperature at each of the eight ROIs (outcomes) and explanatory variables at a 15% significance level was examined using either the Wald test for continuous and binary variables, or the Likelihood Ratio Test (LRT) for categorical variables. Several variables were considered for ADLAs, i.e., had ever experienced ADLAs (yes/no), number of ADLAs in the past month, and in the past 6 months. In the case of highly correlated explanatory variables (correlation coefficient ≥ 0.8), the variable yielding the lowest Akaike Information Criterion (AIC) was selected. The AIC was used to identify the best fitting multivariate model. Marginal estimates of temperature by lymphoedema stage were estimated using the respective mixed-effects multivariate models and the STATA command “margins” and were plotted using “marginsplot”. Differences in temperature across three lymphoedema stages were estimated using the “contrasts” command and plotted with “coeffplot”.

To examine the relationship between (i) the temperatures from each of the eight ROIs, and (ii) the temperature at the calf, and calf compressibility measure, Spearman’s correlation coefficients were performed. Data were stratified by lymphoedema Stage (0–3), i.e., the analysis for each stage was conducted separately. To exclude within-individual correlation for participants who had bilateral lymphoedema, only one leg per person and per stage were included in the analysis. For participants with bilateral lymphoedema and legs at different stage, both legs were included in the analysis in their respective stage group, resulting in one leg per participant per stage-related group. For participants who had both legs at the same stage (*n* = 28), the dominant leg was selected to be included in the analysis. For the only participant who had no leg dominance, the right leg was randomly selected for inclusion. For participants with unilateral lymphoedema, the non-affected leg was included in the “Stage 0” group and the affected leg was included in the group of its respective stage.

### 2.7. Ethics Statement

Ethical approval was obtained from the Liverpool School of Tropical Medicine, Research Ethics Committee (Research Protocol 20–008) and the Bangladesh Medical Research Council, Bangladesh. All participants were adults over the age of 18 years and provided their consent to be included after receiving information on the aims and procedures of this study.

### 2.8. COVID-19 Precautions

This study was conducted during the SARS-CoV-2 pandemic and a risk assessment was implemented before commencement of the field work. The risk was found to be low in the study areas based on national metrics, and communication with district and subdistrict health personnel. The field teams received training on the national Environmental and Social Management Framework for COVID-19 Emergency Response and Pandemic Preparedness, including personal safety procedures and mitigation measures [36]. No adverse events were reported during participant recruitment and data collection.

## 3. Results

### 3.1. Summary

Approximately 10 adults with lower-limb lymphoedema at any level of severity were enrolled in each community clinic. In total, 153 adults (male = 36, 23.5%; female = 117, 76.5%) presented to the clinic and all were included in the survey as they met the inclusion criteria. The mean age of participants was 60 years (male 60.4; female 61.0), and the majority were married (*n* = 95; 62.1%) or widowed (*n* = 56; 36.6%); illiterate (*n* = 127; 83%) and not in paid employment outside the home (*n* = 123; 80.4%).

Almost half (*n* = 75, 49%) had unilateral lymphoedema. There was no lymphedema present (Stage 0) on 38 right legs and 37 left legs. On the affected side, 10 legs (13.3% of unilateral lymphoedema cases) had mild lymphoedema (Stage 1), 23 legs (30.1%) were moderate (Stage 2) and 42 legs (56%) were affected by severe lymphoedema (Stage 3).

The remaining 78 participants (51%) had bilateral lymphoedema which presented as bilateral mild lymphoedema (Stage 1) in two cases (2.6% of bilateral lymphoedema cases), bilateral moderate lymphoedema (Stage 2) in 12 cases (15.4%), and bilateral severe lymphoedema (Stage 3) in 14 cases (17.9%). There were 50 participants with a different stage of lymphoedema on each leg (64.1%).

Most participants (*n* = 122; 79.7%) reported that they experienced ADLA in the past, with 85 participants (69.7%) reporting at least one ADLA episode in the last month, and 120 (98.4%) reporting at least one in the previous six months.

### 3.2. Temperature Visualisation

**Figure 2:**
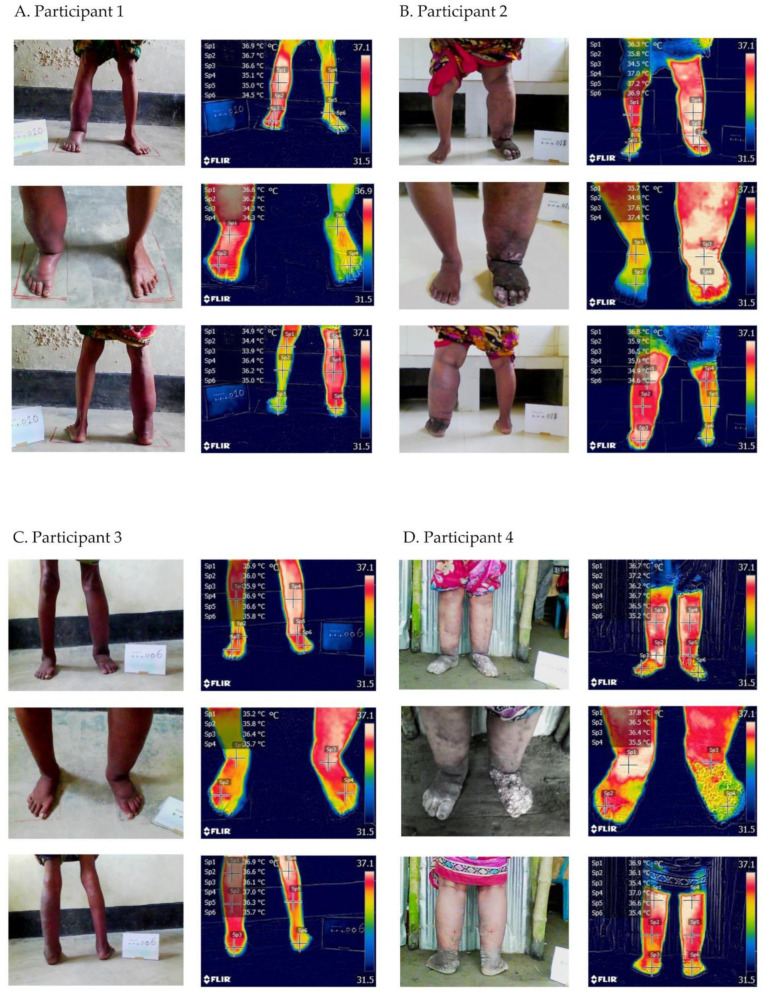
Cases studies–images and temperature measurements from four participants (**A**–**D**) with lymphoedema. Each participant had three images taken including the whole lower-leg (front and back), and a close-up image of the ankle and toes.

To demonstrate how the temperature visualisation was performed, four case studies were included, two participants with unilateral lymphoedema and two participants with bilateral lymphoedema as shown in Figure 2A–D (see online Figure S2 for larger images).

#### 3.2.1. Participant 1

Participant 1: Female, 65 years old, illiterate, married, not in paid employment, unilateral right-leg lymphoedema, Stage 2 (moderate). Temperatures on the affected leg were higher (range 35.0–36.7 °C) than on the unaffected leg (range 33.9−35.1 °C) leg (Figure 2A). The circumference of the affected leg was 1.43-fold greater than the unaffected leg, and the between leg difference in Indurometer values was 8% (Box 1).

Box 1 **Characteristics of participant 1.**

Right leg—moderate lymphoedema (Stage 2) with shallow skin folds
  ⚬ Temperatures—shin 36.9 °C, ankle 36.7 °C, foot 36.6 °C, close ankle 36.6 °C, close foot 36.2 °C, back knee 36.4 °C, calf 36.2 °C, heel 35.0 °C
  ⚬ Circumference 33.0 cm and compressibility 2.96
Left leg—no lymphoedema (Stage 0)
  ⚬ Temperatures—shin 35.1 °C, ankle 35.0 °C, foot 34.5 °C, close ankle 34.3 °C, close foot 34.3 °C, back knee 34.9 °C, calf 34.4 °C, heel 33.9 °C
  ⚬ Circumference 23 cm and compressibility 3.995
ADLA presence, one in last month, three in last six months
Room temperature 33.7 °C; room humidity 55%

#### 3.2.2. Participant 2

Participant 2: Female, 55 years old, illiterate, married, not in paid employment, unilateral left-leg lymphoedema, Stage 3 (severe). Temperatures on the affected leg were higher (range 36.4–37.6 °C) than on the unaffected leg (range 33.9–36.3 °C) (Figure 2B). The circumference of the affected leg was 1.6-fold greater than the unaffected leg, and the between leg difference in Indurometer values was 11% (Box 2).

Box 2 **Characteristics of participant 2.**

Right leg—no lymphoedema (Stage 0)
  ⚬ Temperatures—shin 36.3 °C. ankle 35.7 °C, foot 34.3 °C, close ankle 35.7 °C, close toes 33.9 °C, back knee 35.9 °C, calf 34.9 °C, heel 34.6 °C
  ⚬ Circumference 28.5 cm and compressibility 3.315
Left leg—severe lymphoedema (Stage 3) with deep skin folds, skin knobs and mossy lesions
  ⚬ Temperatures—shin 37.0 °C, ankle 37.2 °C, foot 36.6 °C, close ankle 37.6 °C, close toes 36.4 °C, back knee 36.8 °C, calf 35.9 °C, heel 36.5 °C
  ⚬ Circumference 46.0 cm and compressibility 3.68
ADLA presence, one in last month, three in last six months
Room temp 30.5 °C, room humidity 80%

#### 3.2.3. Participant 3

Participant 3: Male, 72 years old, illiterate, married, not in paid employment, bilateral lymphoedema at Stage 1 (mild) on the right leg and Stage 2 (moderate) on the left leg. Temperatures on the moderate leg (Stage 2; range 35.8–36.9 °C) were similar to the mild leg (Stage 1; range 35.2–37.0 °C) (Figure 2C). The moderate leg circumference was 1.38-fold greater than the mild leg and the between leg difference in Indurometer values was 28% (Box 3).

Box 3 **Characteristics of participant 3.**

Right leg—mild lymphoedema (Stage 1)
  ⚬ Temperatures—shin 35.9 °C; ankle 36.0 °C; foot 35.9 °C, close ankle 35.2 °C; close foot 35.8 °C; back knee 37.0 °C, calf 36.3 °C, heel 35.7 °C
  ⚬ Circumference 25.7 cm and Compressibility 2.81
Left leg—moderate lymphoedema (Stage 2) with shallow skin folds
  ⚬ Temperatures—shin 36.9 °C; ankle 36.6 °C, foot 35.8 °C, close ankle 36.4 °C, close foot 35.7 °C, back knee 36.9 °C, calf 36.6 °C, heel 36.1 °C
  ⚬ Circumference 35.5 cm and Compressibility 2.025
ADLA presence, one in last month, eight in last six months
Room temp 32.3 °C, room humidity 70%

#### 3.2.4. Participant 4

Participant 4: Female, 60 years old, illiterate, married, not in paid employment, bilateral lymphoedema at Stage 3 (severe) on both legs. Temperatures ranges were similar for both legs (right leg range 35.4–37.8 °C, left leg range 35.4–36.9 °C). However, the temperatures were lower on the left toes affected by knobs and mossy lesions (35.5 °C vs. 36.5 °C) (Figure 2D). Leg circumferences and tissue compressibility were similar on both legs (Box 4).

Box 4 **Characteristics of participant 4.**

Right leg—severe lymphoedema (Stage 3)
  ⚬ Temperatures—shin 36.7 °C; ankle 37.2 °C; foot 36.2 °C, close ankle 37.8 °C, close foot 36.5 °C, back knee 37.0 °C, calf 36.6 °C, heel 35.4 °C
  ⚬ Circumference 41.0 cm and Compressibility 2.470
Left leg—severe lymphoedema (Stage 3) with deep ankle folds, skin knobs and mossy lesions
  ⚬ Temperatures—shin 36.7 °C; ankle 36.5 °C, foot 35.2 °C, close ankle 36.4 °C; close foot 35.5 °C, back knee 36.9 °C, calf 36.1 °C, heel 35.4 °C
  ⚬ Circumference 40.0 cm and Compressibility 2.475
ADLA presence, one in last month, one in last six months
Room temp 29.1 °C, room humidity 84%

### 3.3. Temperature Differences at Each Region of Interest (ROI) by Lymphoedema Stage

Overall, the temperature measurements increased by stage with the greatest differences between no lymphoedema (Stage 0, range 33.9–34.9 °C) and severe lymphoedema (Stage 3, range 34.8−35.7 °C). All temperature data are given in Table S1.

**Figure 3:**
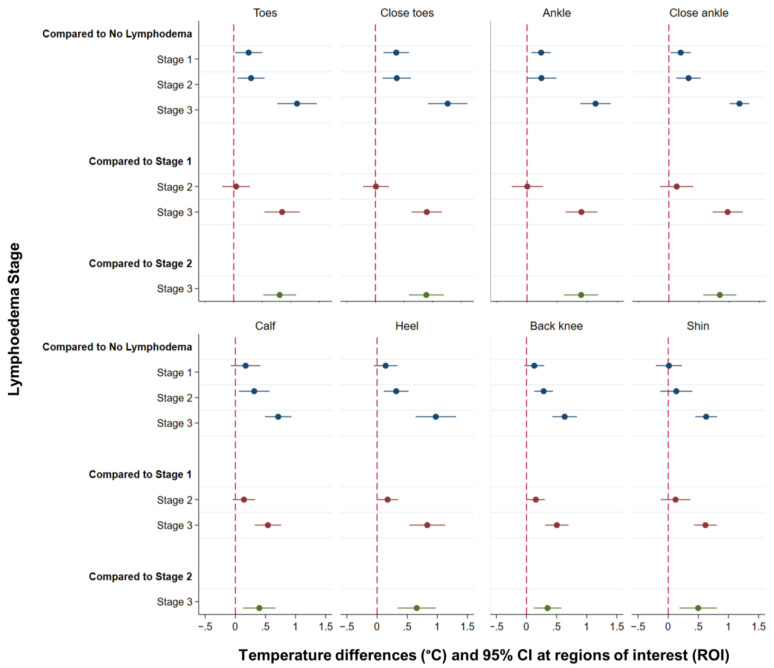
Comparison of temperature differences at regions of interest (ROIs) by lymphoedema stage. First section: No lymphoedema (Stage 0) compared to mild, moderate, severe (Stages 1–3); second section: mild (Stage 1) compared to moderate and severe lymphoedema (Stages 2 and 3), and third section: moderate (Stage 2) compared to severe lymphoedema (Stage 3). The red dashed line indicates a coefficient value of zero. Therefore, a coefficient which confidence interval (CI) crosses the dash/zero line is not significant at 95% level.

Figure 3 graphs the difference in temperature between all stages of lymphoedema as estimated by the mixed multivariate regression model adjusting for room temperature (see figure note) and the estimated differences in temperature, 95% CI and *p*-values are given in Table 1A–C.

Figure 3, Section 1 shows that temperatures measured at the toes, close toes, ankle and close ankle were able to discriminate legs with mild lymphoedema (Stage 1) from those with no lymphoedema (Stage 0), who had significantly lower temperatures (difference range: 0.20–0.37 °C; Table 1A).

Additionally, temperatures measured at all sites except at the ankle and at the shin were significantly different between moderate (Stage 2) and no lymphoedema (Stage 0) (difference range: 0.29–0.37 °C; Table 1A). Severe lymphoedema (Stage 3) had significantly higher temperatures than no lymphoedema (Stage 0) at all sites (difference range: 0.63 °C–1.27 °C; Table 1A).

The second section of Figure 3 shows that differences in temperature between mild lymphoedema (Stage 1) and moderate lymphoedema (Stage 2) were significant only when measured at the heel and the back knee (differences 0.16 °C and 0.18 °C, respectively; Table 1B).

Finally, that all measures were significantly higher in people with severe lymphoedema compared to mild lymphoedema (Figure 3, Section 2; difference range: 0.50–0.97 °C; Table 1B) and moderate lymphoedema (Figure 3, Section 3; difference range: 0.35–0.90 °C; Table 1C).

### 3.4. Temperature Differences at Each Region of Interest (ROI) by ADLA History

Leg temperatures were lower among participants who had no history of ADLA (range 33.0–35.3 °C) than for participants who reported any past ADLA (range 34.1–35.9 °C). The greatest differences between participants with or without a history of ADLA were observed on unaffected (Stage 0) legs (no ADLA range 33.0–34.6 °C, previous ADLA range 34.1–35.0 °C) and on legs affected by severe lymphoedema (Stage 3), (no ADLA range 34.0–35.3 °C, previous ADLA range 35.1–35.9 °C). See Table S2 for all temperature data by ADLA status and lymphoedema stage.

Our multivariate models adjusting for room temperature estimated that temperatures were significantly higher at every measurements site on the legs of participants who had experienced any past ADLA, compared to those who had not, and regardless of lymphoedema stage (coefficient range 0.49–0.82) (Table 2).

**Figure 4:**
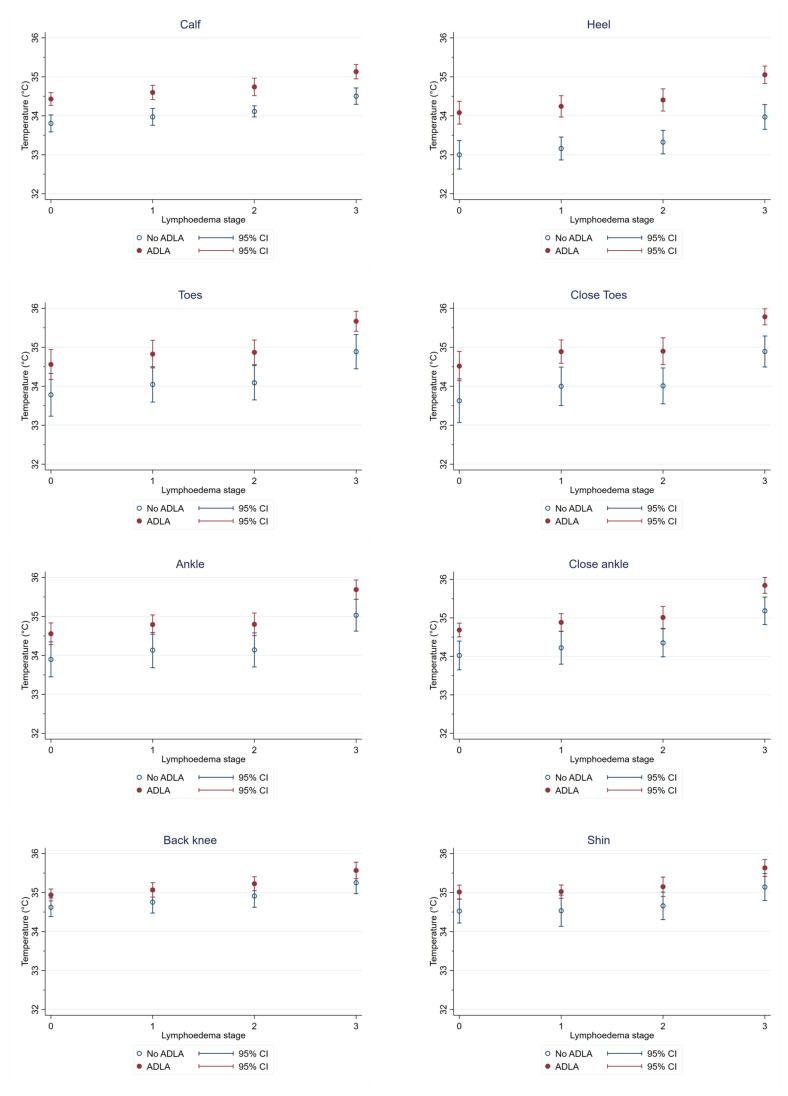
Comparison of temperatures at regions of interest (ROIs) by history of ADLA and lymphoedema stage.

Figure 4 shows the estimated temperature for each ROI by ADLA status and lymphoedema stage. Temperatures measured at the heel and calf appeared to be the most robust to differentiate patients with a history of ADLA vs. no history of ADLA, as they were the only measures that identified significant differences between patients with a history of ADLA and no history of ADLA at all lymphoedema stages (Table S2). Temperature measures were also significantly higher among participants with a history of ADLA at the toes, ankle and close ankle for Stage 3, and at the close ankle for Stage 0.

### 3.5. Correlation between Temperature Measures at Different Regions of Interest (ROIs)

Significant positive correlations were found between all ROIs in all lymphoedema stages (0–3). The highest correlations were between the ankle and toes for legs with no or moderate lymphoedema (Stage 0, and 1; correlation coefficient range 0.895–0.89), between the toes and heel for mild lymphoedema (Stage 1; correlation coefficient 0.88), and at the back of the knee and the calf for severe lymphoedema (Stage 3; correlation coefficient 0.906) (see Table S3 for Spearman’s correlation coefficient data).

### 3.6. Correlation between Objective Outcome Measures

Significant weak negative correlations were found between temperature measures at the calf and the mid-calf circumference of legs with no lymphoedema (Stage 0; correlation coefficient −0.275), and with the mid-calf tissue compressibility of legs with mild and severe lymphoedema (Stage 1; correlation coefficient −0.282, Stage 3; correlation coefficient −0.298) (see Table S4 for Spearman’s correlation coefficient data).

## 4. Discussion

This study demonstrates the potential of thermal imaging cameras as a non-invasive point-of-care tool for researchers, programme managers and local health workers in endemic areas to assess and monitor filarial lymphoedema. The FLIR C3 camera used in this study is compact, portable, robust, simple to use [34] and suitable for field work in remote and rural areas where environmental conditions can be extreme.

The surface temperature of affected limbs increased with the severity of lymphoedema across all anatomical sites, although not always significantly. Temperatures were further elevated across all sites in patients with experience of ADLA. In bilateral lymphoedema, both legs had similar temperatures. However, lower temperatures were recorded in ROIs where skin pathologies and tissue fibrosis form knobs and mossy lesions where the thickened tissue structures may have reduced blood flow [23,37]. Such feature-related imagery would enable health workers to identify and provide targeted intervention and support, as well as objectively monitor temperatures changes which may reflect improvements in blood circulation to the affected area. The availability of thermal cameras at selected community clinics and/or referral centres would allow for rapid evaluation to guide appropriate treatment and training in self-care. Baseline measures recorded for individuals would enable monitoring of the effectiveness of care-packages and other interventions, and tailoring of individual self-care recommendations over time.

The significant temperature differences found between legs with no lymphoedema and mild lymphoedema suggest that thermal imaging may be able to detect subclinical lymphoedema by identifying temperature hotspots in unaffected legs without other clinical presentation. A similar approach may be implemented to what has been demonstrated with the Indurometer in asymptomatic young people in Myanmar [38]. The detention of subclinical tissue and vascular changes in diseases such as diabetes has been previously reported [26,39], and the obvious changes at the toes and ankle visualised in this study, indicate that thermal images at these key sites maybe an effective and efficient way to monitor for new cases. The interdigital spaces of the toes and ankle skin folds are commonly affected by bacterial and fungal infections through breaks in the skin such as wounds, cuts, lesions that become inflamed if not protected with shoes or kept clean through washing [3,40,41,42]. Interdigital entry lesions have been identified as an important risk factor for episodes of ADLAs [40] and thermal imagery to identify these at risk regions, offers the opportunity to establish appropriate self-care practices to prevent progression to clinical disease.

Overall, temperatures increased with an increase in lymphoedema stage as did the areas of the leg involved. Subclinical and mild changes appear first at the toes and ankle, and in moderate lymphoedema the affected areas extended to include the heel and knee, with all measurement points involved in severe lymphoedema. The significantly higher temperatures in severely affected legs is of particular concern as they are also more likely to suffer from ADLA [10,43], which was also associated with higher temperatures. For the most severely affected patients this could result in an increase in the frequency and intensity of pain [44], and reduce their mobility and quality of life [33,45].

The temperatures for all ROIs were correlated, with the most positive correlations between the toes, ankle and heel, for legs with no, mild and moderate lymphoedema progressively. The correlations between the posterior knee and calf sites for legs with severe lymphoedema suggest that these sites may be similarly affected due to the underlying lymphatic pathway and that a single site at the posterior knee may be sufficient to assess progression between moderate and severe stages.

When tissue compressibility measures are considered, they suggest that legs with higher temperature have undergone more pathological changes than unaffected or mild stage legs. However, unlike the Indurometer, thermal images in this study presented a clearly linear relationship between measures, which can be easily interpreted by local health staff.

The main limitation to this study was related to the FLIR C3 Compact Thermal Imaging Camera lower resolution compared to the Android-based thermal imaging camera attached to an iPhone (FLIR ONE Pro), or high-end infrared device by Thermo Tracer TH700N, Nippon Avionics Co., Ltd., Tokyo, Japan), which have been validated [26]. The use of the iPhone (FLIR ONE) was piloted for filarial lymphoedema in Malawi in 2019. However, given the FLIR C3′s robustness and practicality for health workers in rural field settings, the authors decided that these benefits outweighed the more fragile and expensive options. A comparison of cameras should be considered in future studies as different camera models may be suitable in different settings and for specific research questions. Further limitations were related to (i) not including a control group of unaffected people, i.e., no lymphoedema, to determine whether temperatures compare to a Stage 0 leg in an affected person with unilateral lymphoedema; (ii) only one thermal image of the lower limbs (front and back) and close-up of ankle and toes being taken per participate, thus limiting comparisons between images; and (iii) the temperature ROI on the mid-calf could not be exactly matched with the site of the tissue compressibility, which may have influenced the relationship between these two measures.

## 5. Conclusions

This study has shown that thermal imaging is an innovative tool able to objectively measure the status of lower-limb filarial lymphoedema. This offers the potential to establish baseline measures for individuals, which can be used to monitor lymphoedema progression or the effects of interventions [32,33]. Such a portable and non-invasive tool is needed by health workers in LF-endemic regions [16,17], and may have use for filarial hydrocoele [46] or for other skin NTDs such as onchocerciasis, podoconiosis and leprosy, which could also help in differential diagnosis in co-endemic areas [8,32,47,48,49]. The use of thermal imaging requires minimal training, and the results can be easily and immediately visualised and interpreted to guide ongoing lymphoedema detection and management at the point of care. The use of this tool will optimise long-term care for people affected by lymphoedema, and improve the capacity of primary health care workers to deliver the essential package of care as part of universal health coverage and the WHO NTD Road Map for 2021–2030 [1].

## Figures and Tables

**Table 1:** Difference in temperature between lymphoedema stages.

**(A) Compared to No Lymphoedema (Stage 0)**			
	**Temperature** **difference °C**	**95% CI**	***p*-value**
**Mild (Stage 1)**			
Toes	0.26	0.02–0.50	0.033 *
Close toes	0.37	0.14–0.59	0.001 *
Ankle	0.23	0.07–0.40	0.005 *
Close ankle	0.20	0.03–0.36	0.022 *
Calf	0.17	−0.07–0.42	0.17
Heel	0.14	−0.06–0.34	0.159
Back knee	0.13	−0.03–0.29	0.119
Shin	0.01	−0.21–0.23	0.916
**Moderate (Stage 2**)			
Toes	0.30	0.07–0.54	0.012 *
Close toes	0.37	0.13–0.62	0.003 *
Ankle	0.24	−0.01–0.49	0.058
Close ankle	0.33	0.12–0.53	0.002 *
Calf	0.31	0.06–0.57	0.016 *
Heel	0.32	0.11–0.52	0.002 *
Back knee	0.29	0.13–0.44	<0.0001 *
Shin	0.13	−0.13–0.40	0.327
**Severe (Stage 3)**			
Toes	1.11	0.76–1.46	<0.0001 *
Close toes	1.27	0.92–1.61	<0.0001 *
Ankle	1.14	0.89–1.39	<0.0001 *
Close ankle	1.17	1.01–1.33	<0.0001 *
Calf	0.71	0.49–0.93	<0.0001 *
Heel	0.97	0.64–1.31	<0.0001 *
Back knee	0.63	0.43–0.84	<0.0001 *
Shin	0.63	0.45–0.81	<0.0001 *
**(B) Compared to Mild Lymphoedema (Stage 1)**			
**Moderate (Stage 2)**			
Toes	0.04	−0.20–0.29	0.735
Close toes	0.01	−0.22–0.24	0.94
Ankle	0.01	−0.25–0.26	0.971
Close ankle	0.13	−0.15–0.41	0.357
Calf	0.14	−0.04–0.33	0.135
Heel	0.18	0.004–0.35	0.045 *
Back knee	0.16	0.005–0.31	0.043 *
Shin	0.12	−0.12–0.37	0.329
**Severe (Stage 3)**			
Toes	0.85	0.54–1.16	<0.0001 *
Close toes	0.90	0.63–1.17	<0.0001 *
Ankle	0.90	0.64–1.16	<0.0001 *
Close ankle	0.97	0.72–1.23	<0.0001 *
Calf	0.54	0.32–0.76	<0.0001 *
Heel	0.83	0.54–1.13	<0.0001 *
Back knee	0.50	0.31–0.70	<0.0001 *
Shin	0.62	0.43–0.81	<0.0001 *
**(C) Compared to Moderate Lymphoedema (Stage 2)**			
**Severe (Stage 3)**			
Toes	0.81	0.52–1.09	<0.0001
Close toes	0.89	0.59–1.20	<0.0001
Ankle	0.90	0.61–1.18	<0.0001
Close ankle	0.84	0.57–1.12	<0.0001
Calf	0.40	0.13–0.67	0.004
Heel	0.66	0.34–0.97	<0.0001
Back knee	0.35	0.12–0.58	0.003
Shin	0.50	0.19–0.81	0.002

* Significant at 5% level. CI: confidence interval; lymphoedema stages refer to the WHO staging system. Stage 1: mild; Stage 2: moderate; Stage 3: severe. Results were obtained from multivariate mixed-effects linear regression models adjusting for room temperature and including an individual-level and a village-level random effect.

**Table 2:** Increase in temperature at eight regions of interest (ROIs) in participants with a history of ADLA compared to participants who had never had ADLA.

ADLA	Coefficient	95% CI	*p*-Value
Toes	0.56	0.06–1.06	0.028 *
Close toes	0.68	0.21–1.15	0.005 *
Ankle	0.54	0.10–0.97	0.015 *
Close ankle	0.58	0.17–0.99	0.006 *
Calf	0.53	0.32–0.74	<0.0001 *
Heel	0.82	0.36–1.28	<0.0001 *
Back knee	0.31	0.07–0.54	0.012 *
Shin	0.49	0.19–0.79	0.001 *

* Significant at 5% level. CI: confidence interval; ADLA: acute dermatolymphangioadenitis; lymphoedema stages refer to the WHO staging system. Stage 1: mild; Stage 2: moderate; Stage 3: severe. Coefficients estimate the increase in temperature (°C) for participants with a history of ADLA compared to those with no history of ADLA. Results were obtained from multivariate mixed-effects linear regression models including an individual-level and a village-level random effect.

## Data Availability

The majority of data presented in this paper are available in the text, related tables or supplementary files. Individual data will be available from the corresponding author on reasonable request and according to IRB restrictions.

## Supplementary Materials

The following are available online at https://www.mdpi.com/article/10.3390/jcm10112301/s1, Figure S1: Camera set up for taking front and back images of participants lower legs. Figure S2: Larger images of case study participants. Table S1: Temperature data related to lymphoedema stage. Table S2: Mean temperature data related to ADLA history by lymphoedema stage. Table S3: Spearman’s correlation coefficients between all eight leg measurements temperatures. Table S4: Spearman’s correlation coefficients between temperature, leg circumference and tissue compressibility.
