# Supplementary material for: Infrared Thermal Imaging as a Novel Non-Invasive Point-of-Care Tool to Assess Filarial Lymphoedema
Source: J Clin Med. 2021 May 25;10(11):2301. doi: 10.3390/jcm10112301 (PMC8198125; doi:10.3390/jcm10112301)
Supplement: Supplementary file 1 [file jcm-10-02301-s001.zip › Additional files/Table S1.pdf]

Table S1. Temperature data related to lymphoedema stage

Measurement Site	Lymphoedema Stage	Mean Temperature	95% CI
**Shin**	0	34.9	34.7 - 35.1
	1	34.9	34.7 - 35.2
	2	35.0	34.8 - 35.3
	3	35.5	35.3 - 35.8
	Mean	35.1	
**Ankle**	0	34.4	34.1 - 34.7
	1	34.7	34.4 - 34.9
	2	34.7	34.4 - 35.0
	3	35.6	35.3 - 35.8
	Mean	34.9	
**Toes**	0	34.4	34.0 - 34.8
	1	34.7	34.3 - 35.0
	2	34.7	34.4 - 35.0
	3	35.5	35.3 - 35.8
	Mean	34.8	
**Close Ankle**	0	34.5	34.3 - 34.8
	1	34.7	34.5 - 35.0
	2	34.9	34.6 - 35.2
	3	35.7	35.5 - 35.9
	Mean	35.0	
**Close Toes**	0	34.3	33.9 - 34.8
	1	34.7	34.4 - 35.0
	2	34.7	34.3 - 35.1
	3	35.6	35.4 - 35.8
	Mean	34.8	
**Back knee**	0	34.9	34.7 - 35.0
	1	35.0	34.8 - 35.2
	2	35.2	35.0 - 35.3
	3	35.5	35.3 - 35.7
	Mean	35.2	
**Calf**	0	34.3	34.1 - 34.5
	1	34.5	34.3 - 34.7
	2	34.6	34.4 - 34.8
	3	35.0	34.8 - 35.2
	Mean	34.6	

**Heel**	0	33.9	33.5 - 34.2
	1	34.0	33.7 - 34.3
	2	34.2	33.9 - 34.5
	3	34.8	34.6 - 35.0
	Mean	34.2	
