# Supplementary material for: Infrared Thermal Imaging as a Novel Non-Invasive Point-of-Care Tool to Assess Filarial Lymphoedema
Source: J Clin Med. 2021 May 25;10(11):2301. doi: 10.3390/jcm10112301 (PMC8198125; doi:10.3390/jcm10112301)
Supplement: Supplementary file 1 [file jcm-10-02301-s001.zip › Additional files/Table S2.pdf]

Table S2. Temperature data related to ADLA status by lymphoedema stage

Measurement site	Temperature	95% CI
**Shin**		
Stage 0, no ADLA	34.52	34.22 - 34.83
Stage 0, ADLA	35.01	34.84 - 35.19
stage 1, no ADLA	34.53	34.13 - 34.94
Stage 1, ADLA	35.02	34.85 - 35.19
Stage 2, no ADLA	34.66	34.31 - 35.01
Stage 2, ADLA	35.15	34.90 - 35.40
stage 3, no ADLA	35.14	34.79 - 35.49
Stage 3, ADLA	35.63	35.42 - 35.85
**Ankle**		
Stage 0, no ADLA	33.90	33.45 - 34.35
Stage 0, ADLA	34.56	34.28 - 34.83
stage 1, no ADLA	34.13	33.68 - 34.59
Stage 1, ADLA	34.79	34.54 - 35.04
Stage 2, no ADLA	34.14	33.7 - 34.58
Stage 2, ADLA	34.80	34.51 - 35.09
stage 3, no ADLA	35.03	34.62 - 35.44
Stage 3, ADLA	35.69	35.44 - 35.93
**Toes**		
Stage 0, no ADLA	33.78	33.23 - 34.33
Stage 0, ADLA	34.56	34.18 - 34.94
stage 1, no ADLA	34.05	33.59 - 34.50
Stage 1, ADLA	34.83	34.47 - 35.18
Stage 2, no ADLA	34.09	33.65 - 34.53
Stage 2, ADLA	34.87	34.55 - 35.19
stage 3, no ADLA	34.89	34.45 - 35.33
Stage 3, ADLA	35.67	35.41 - 35.93
**Close Ankle**		
Stage 0, no ADLA	34.02	33.65 - 34.40
Stage 0, ADLA	34.69	34.51 - 34.86
stage 1, no ADLA	34.22	33.80 - 34.65
Stage 1, ADLA	34.88	34.65 - 35.11
Stage 2, no ADLA	34.35	33.99 - 34.71
Stage 2, ADLA	35.01	34.73 - 35.30
stage 3, no ADLA	35.18	34.83 - 35.54
Stage 3, ADLA	35.85	35.64 - 36.05
**Close Toes**		
Stage 0, no ADLA	33.78	33.23 - 34.33
Stage 0, ADLA	34.56	34.18 - 34.94
stage 1, no ADLA	34.05	33.59

Stage 1, ADLA	34.83	34.47 - 35.18
Stage 2, no ADLA	34.09	33.65 - 34.50
Stage 2, ADLA	34.87	34.55 - 35.19
stage 3, no ADLA	34.89	34.45 - 35.33
Stage 3, ADLA	35.67	35.41 - 35.93
**Back**		
**Back knee**		
Stage 0, no ADLA	34.62	34.38 - 34.86
Stage 0, ADLA	34.94	34.78 - 35.09
stage 1, no ADLA	34.75	34.47 - 35.03
Stage 1, ADLA	35.07	34.88 - 35.25
Stage 2, no ADLA	34.91	34.62 - 35.20
Stage 2, ADLA	35.23	35.05 - 35.41
stage 3, no ADLA	35.25	34.97 - 35.53
Stage 3, ADLA	35.57	35.36 - 35.78
**Calf**		
Stage 0, no ADLA	33.80	33.59 - 34.02
Stage 0, ADLA	34.43	34.27 - 34.60
stage 1, no ADLA	33.97	33.76 - 34.19
Stage 1, ADLA	34.60	34.42 - 34.78
Stage 2, no ADLA	34.11	33.97 - 34.26
Stage 2, ADLA	34.74	34.52 - 34.96
stage 3, no ADLA	34.50	34.29 - 34.71
Stage 3, ADLA	35.13	34.95 - 35.32
**Heel**		
Stage 0, no ADLA	33.00	32.63 - 33.36
Stage 0, ADLA	34.08	33.79 - 34.37
stage 1, no ADLA	33.16	32.87 - 33.46
Stage 1, ADLA	34.24	33.97 - 34.52
Stage 2, no ADLA	33.32	33.02 - 33.63
Stage 2, ADLA	34.41	34.12 - 34.69
stage 3, no ADLA	33.97	33.65 - 34.29
Stage 3, ADLA	35.05	34.83 - 35.28
