# Supplementary figures and images for: Infrared Thermal Imaging as a Novel Non-Invasive Point-of-Care Tool to Assess Filarial Lymphoedema
Source: J Clin Med. 2021 May 25;10(11):2301. doi: 10.3390/jcm10112301 (PMC8198125; doi:10.3390/jcm10112301)

**Figure S1:** Camera set up for taking images of participants lower legs

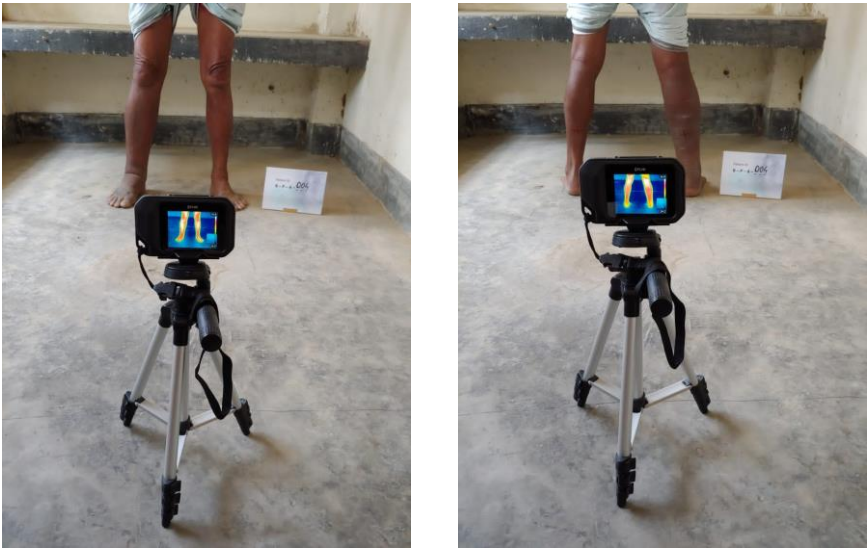

Supplement: Supplementary file 1 [file jcm-10-02301-s001.zip › Additional files/Figure S1.pdf]

Figure S2: Figure 2 - larger images

Participant 1.

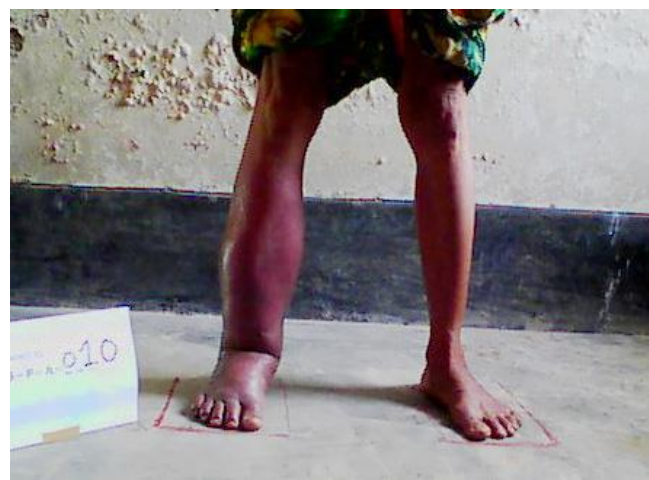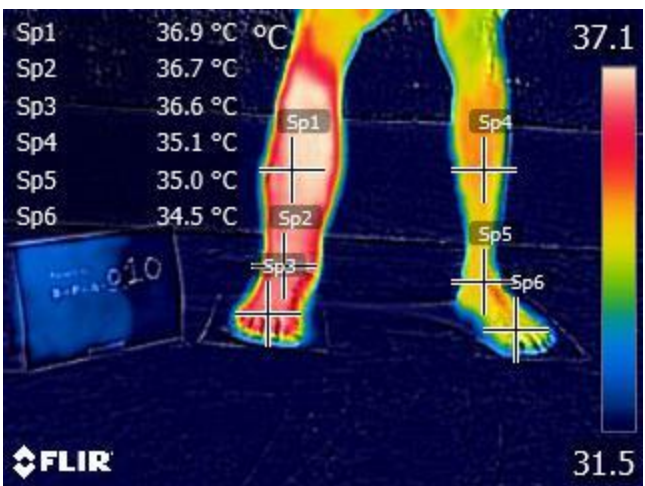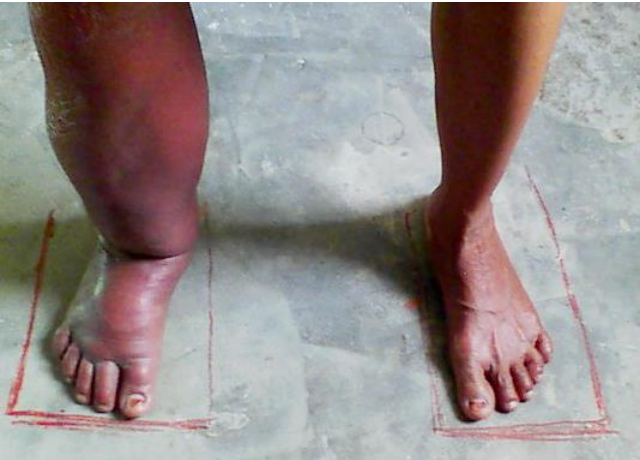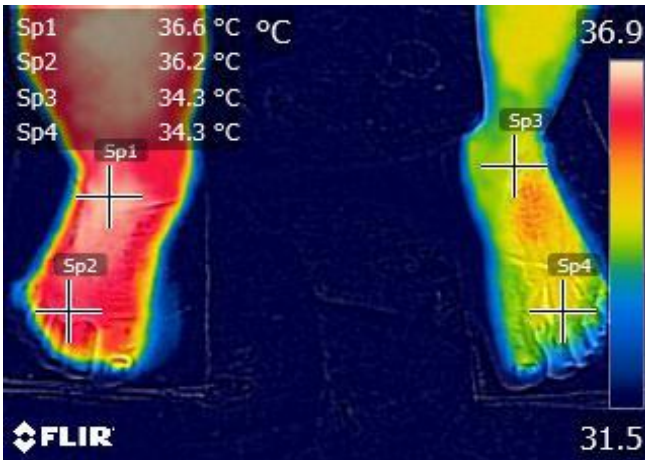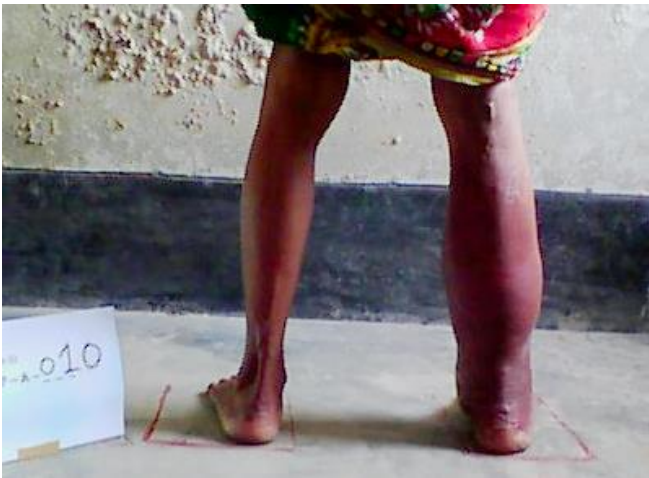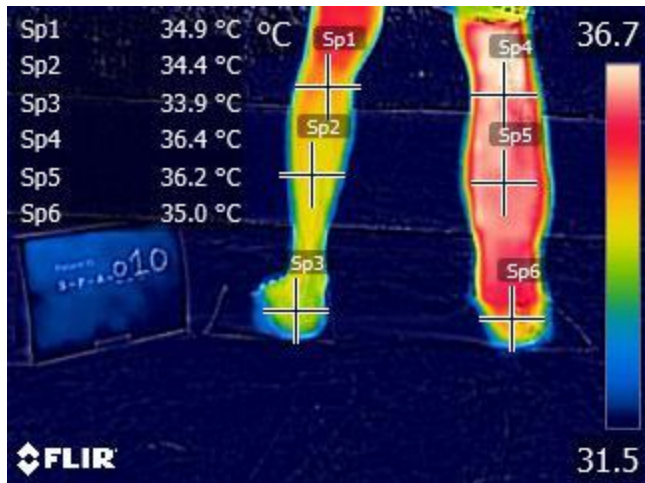

Participant 2.

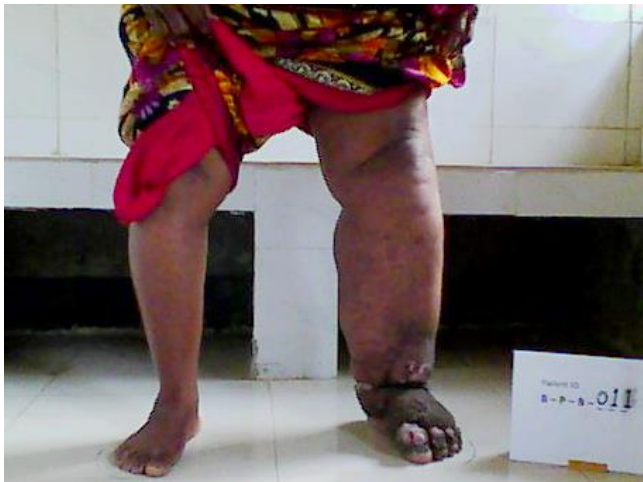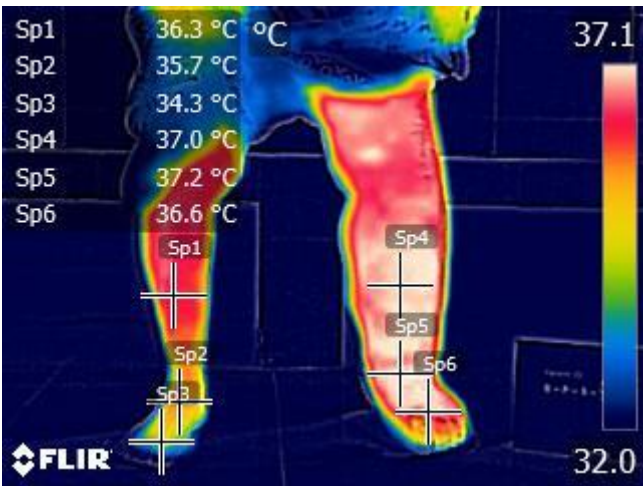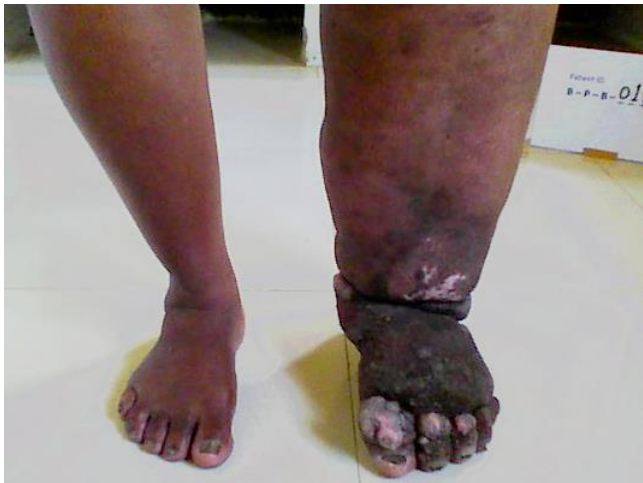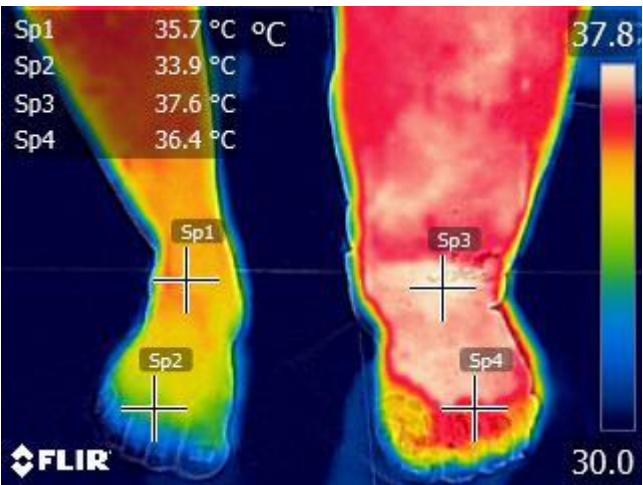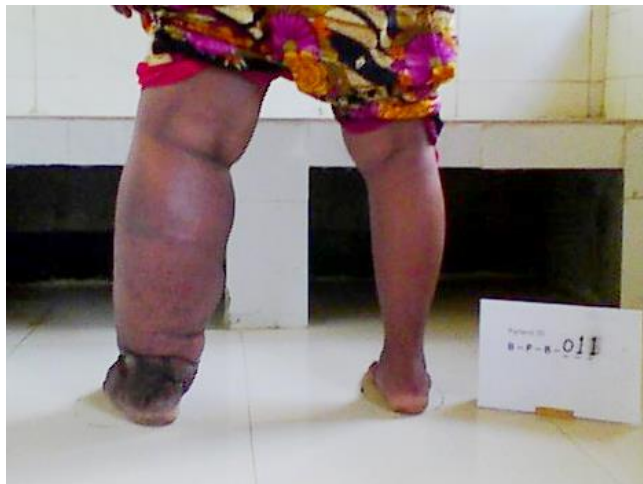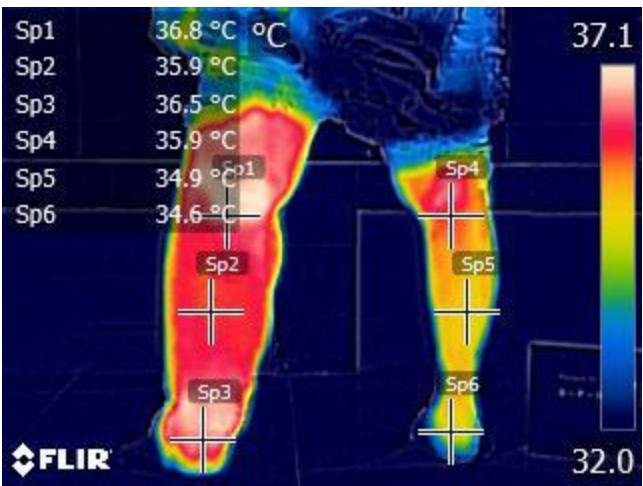

Participant 3.

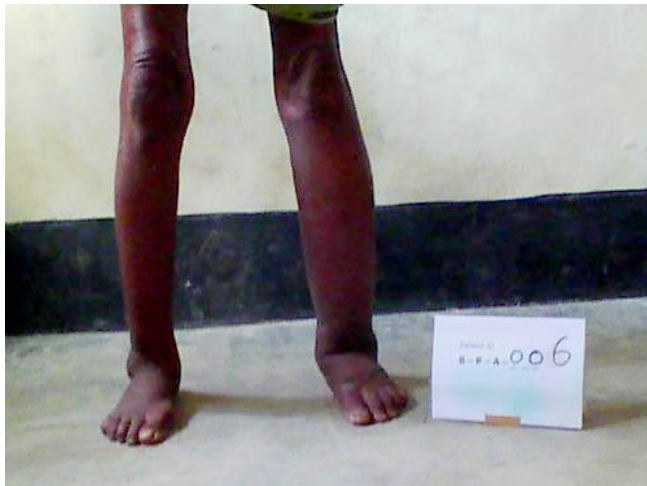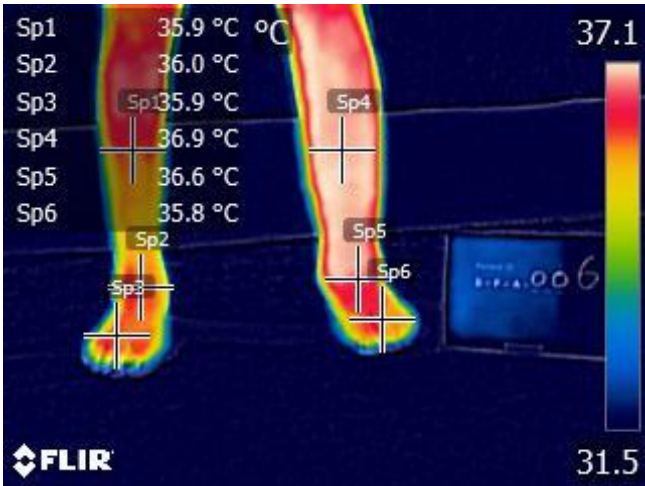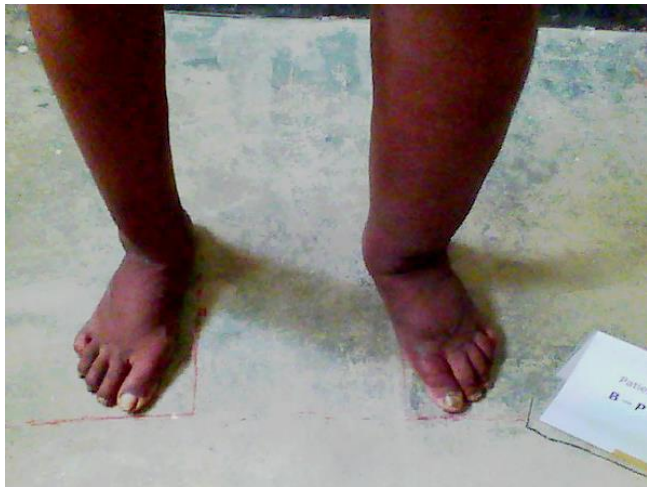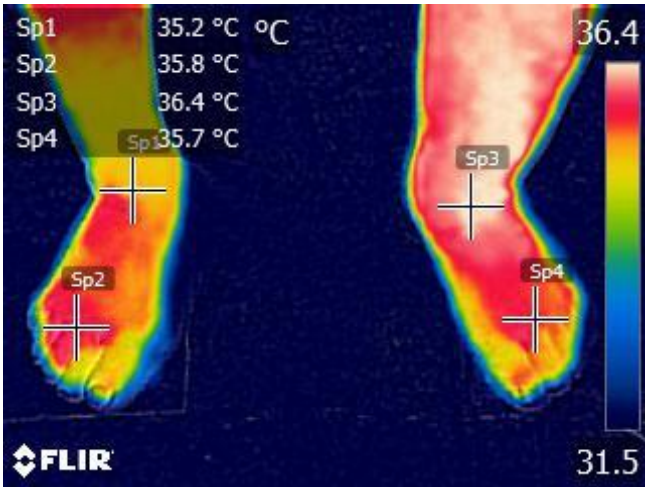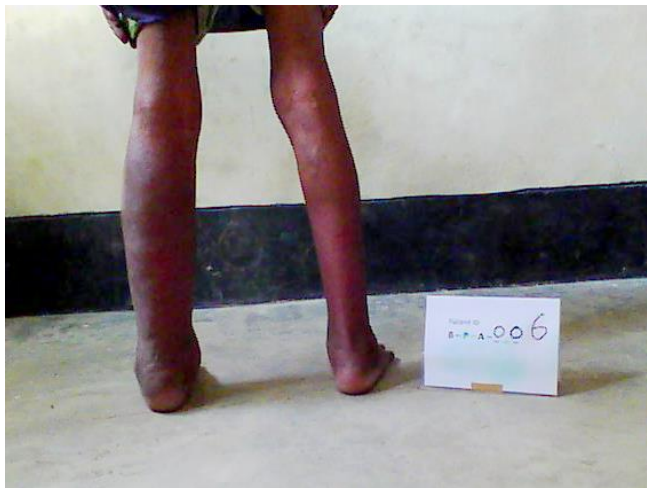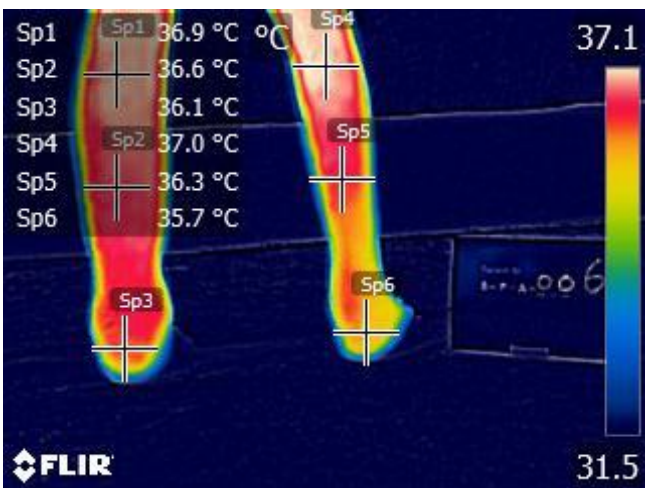

Participant 4.

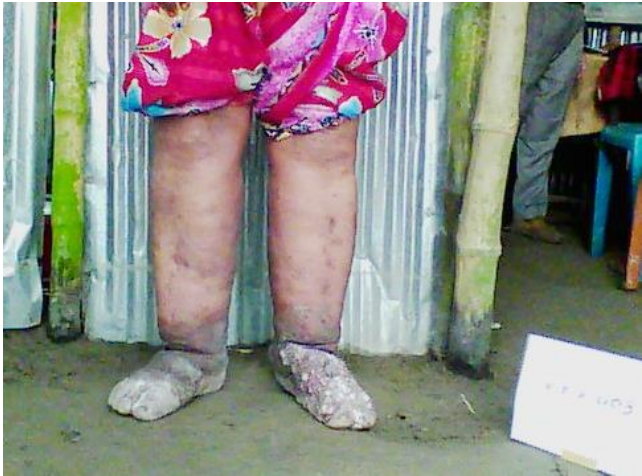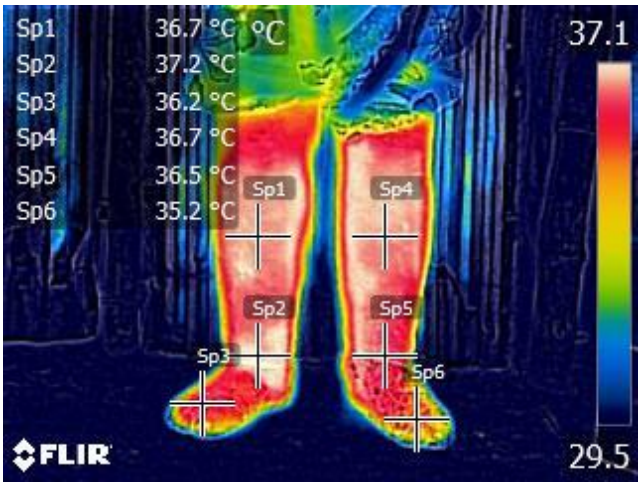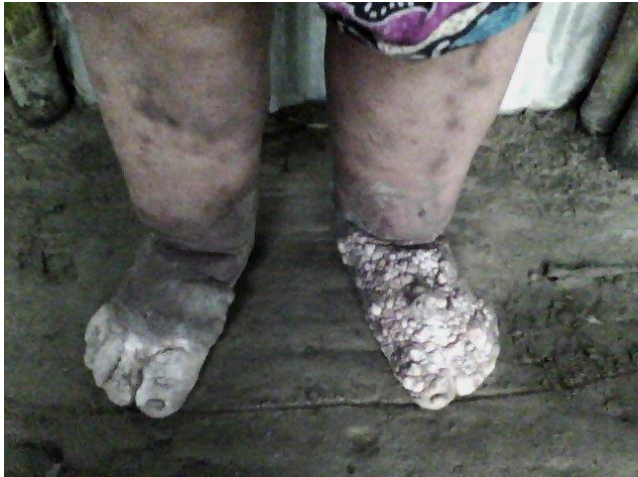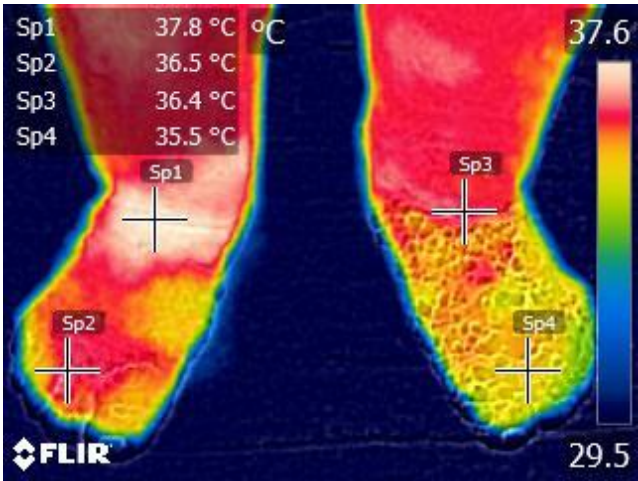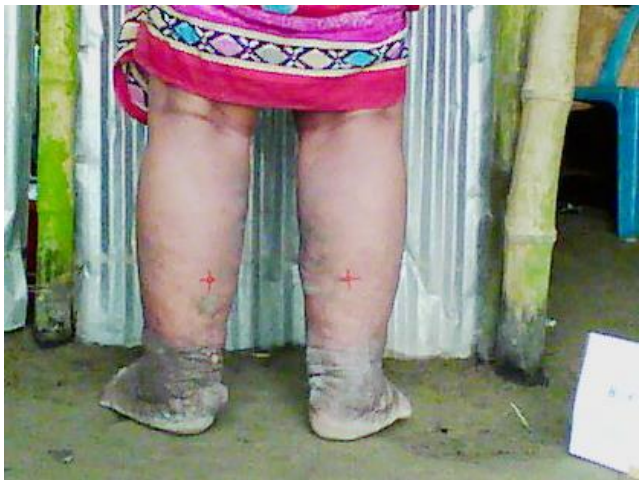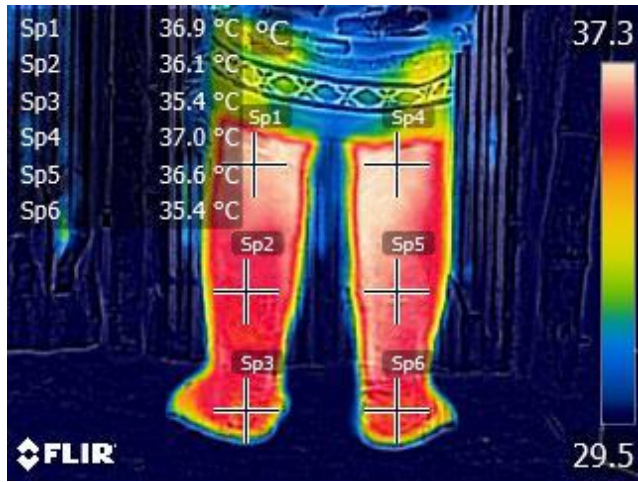

Supplement: Supplementary file 1 [file jcm-10-02301-s001.zip › Additional files/Figure S2.pdf]
